# Using Cholinesterases and Immobilized Luminescent Photobacteria for the Express-Analysis of Mycotoxins and Estimating the Efficiency of Their Enzymatic Hydrolysis

**DOI:** 10.3390/toxins13010034

**Published:** 2021-01-06

**Authors:** Elena Efremenko, Olga Maslova, Nikolay Stepanov, Anvar Ismailov

**Affiliations:** 1 Faculty of Chemistry, Lomonosov Moscow State University, Lenin Hills 1/3, 119991 Moscow, Russia; olga-still@mail.ru (O.M.); na.stepanov@gmail.com (N.S.); 2 N.M. Emanuel Institute of Biochemical Physics RAS, Kosigina str., 4, 119334 Moscow, Russia; 3 Faculty of Biology, Lomonosov Moscow State University, Lenin Hills 1/12, 119234 Moscow, Russia; anvaris@list.ru

**Keywords:** mycotoxins, bioluminescent bacteria, immobilized cells, cholinesterase-based analysis, analytical characteristics, enzymatic detoxification

## Abstract

Novel sensitive analytical agents that can be used for simple, affordable, and rapid analysis of mycotoxins are urgently needed in scientific practice, especially for the screening of perspective bio-destructors of the toxic contaminants. We compared the characteristics of a rapid quantitative analysis of different mycotoxins (deoxynivalenol, ochratoxin A, patulin, sterigmatocystin, and zearalenone) using acetyl-, butyrylcholinesterases and photobacterial strains of luminescent cells in the current study. The best bioindicators in terms of sensitivity and working range (μg/mL) were determined as follows: *Photobacterium* sp. 17 cells for analysis of deoxynivalenol (0.8–89) and patulin (0.2–32); *Photobacterium* sp. 9.2 cells for analysis of ochratoxin A (0.4–72) and zearalenone (0.2–32); acetylcholinesterase for analysis of sterigmatocystin (0.12–219). The cells were found to be more sensitive than enzymes. The assayed strains of photobacterial cells ensured 44%–83% lower limit of detection for deoxynivalenol and sterigmatocystin as compared to the previously known data for immobilized luminescent cells, and the range of working concentrations was extended by a factor of 1.5–3.5. Calibration curves for the quantitative determination of patulin using immobilized photobacteria were presented in this work for the first time. This calibration was applied to estimate the enzyme efficiency for hydrolyzing mycotoxins using zearalenone and His_6_-tagged organophosphorus hydrolase as examples.

## 1. Introduction

Studying mycotoxins is topical and relevant for ensuring food and biological safety [1,2,3]. Analytical approaches to the detection and identification of mycotoxins [4] are being actively developed, and so are strategies for mycotoxin control and detoxification [5,6,7,8].

These are currently the most actively pursued areas: (i) elucidating the mechanisms of toxic effects of mycotoxins on living organisms [5]; (ii) development and testing of effective selective and sensitive analytical methods for the detection of mycotoxins in food, agricultural feed, and raw materials for the pharmaceutical industry [9,10]; (iii) search for sorbents capable of removing mycotoxins from raw materials [11]; (iv) the search for new methods for the destruction of mycotoxins, especially those involving bio-destructors [12,13]. The last of the above directions is especially important today, since it implies the development of means, including combined action, that eliminate mycotoxins not only due to their sorption, but also due to their catalytic decomposition. Various enzymes are considered as such biocatalytic detoxifiers [13,14].

In fundamental and applied research in the field of mycotoxins, liquid chromatography (LC) and enzyme-linked immunosorbent assay (ELISA) are most widely used today. An LC run is followed by mass spectrometry (MS), sensitive fluorescence detection (FLD), or ultraviolet (UV) detection [9,10,12]. High analytical accuracy and selectivity are the main advantages of the abovementioned methods. However, in this case, the duration and complexity of the sample preparation are obvious limiting factors for the express use of LC. ELISA kits allow for express analysis; however, they are distinguished by their high cost, since special antibodies are required for each mycotoxin.

When developing approaches to detoxification of mycotoxins using biocatalysts, selectivity and accuracy are not always priority indicators at the stage of screening and selection of primary candidates. Analytical express methods are more popular at this stage, because they allow the rapid assessment the residual toxicity of the test samples after their enzymatic or cellular treatment. Thus, ineffective candidates can be quickly eliminated from the study, whereas more promising biocatalysts can be chosen for a deeper study of their characteristics using more accurate analytical instruments based on LC or ELISA.

The initial choice of perspective biocatalysts for in-depth study is based on the published data. For example, it is known that enzymes of some classes, including hexa-histidine-tagged organophosphorus hydrolase (His_6_-OPH), exhibit destructive activity towards various mycotoxins [3,9,10,13,14,15,16,17]. Computer design, and, in particular, the molecular docking method [18,19], is yet another technique which has been proved useful for the initial selection of promising potential candidates from a number of enzymes for the decomposition of mycotoxins. The next stages of research already imply practical experimental research.

To quickly screen out candidates selected as a result of docking, but which do not efficiently detoxify mycotoxins, luminescent photobacterial cells can be successfully used for the rapid assessment of the toxicity of samples [17]. These cells sensitively react to the presence of mycotoxins via changing the level of their bioluminescence. It is important that when the cells are used in an immobilized form, such analyzes become possible both in discrete and continuous modes and the analytical signal is stable enough [17,20]. It appears possible to find ways of increasing the sensitivity of mycotoxin detection with this technique by varying the strains of photobacteria immobilized by the same method. We have not identified such comparative studies conducted earlier.

The search for other sensitive analytical agents that can be used for affordable rapid analysis essential for controlling mycotoxins is of obvious scientific and practical importance. In particular, cholinesterases can be considered among the promising candidates, which have proven themselves well in the rapid analysis of many other toxic compounds [21]. Our previous results showing mycotoxins docking to the surface of cholinesterases [13] indicated that inactivation of these enzymes under the action of mycotoxins is feasible. Therefore, cholinesterases (acetyl- (AChE) and/or butyrylcholinesterase (BChE)) can be used for assessing the concentrations of mycotoxins and the effectiveness of the action of destructors on these substances. However, we were unable to find any reports on systematic studies of the inhibition of the cholinesterases by various mycotoxins and on the possibility of implementing an analytical technique based on this effect.

The aim of the present work was to compare the characteristics of cholinesterases and luminescent photobacterial strains for use in rapid quantitative analysis of mycotoxins using cholinesterases and luminescent photobacterial strains. We also studied the applicability of this technique for assessing the effectiveness of mycotoxin biodegradation in the case of zearalenone and the His_6_-OPH.

## 2. Results

### 2.1. The Quantitative Express-Analysis of Mycotoxins in Liquid Media Involving Cholinesterases or Immobilized Bioluminescent Photobacterial Cells

**Figure 1:**
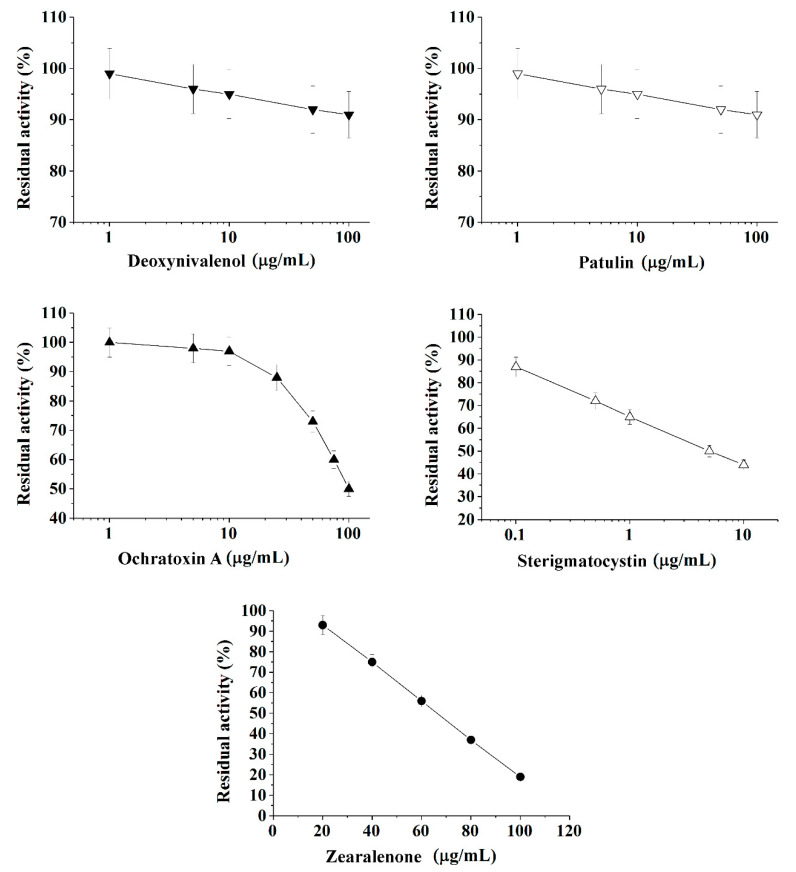
Inhibition effect of deoxynivalenol, patulin, ochratoxin A, sterigmatocystin, and zearalenone on acetylcholinesterase (AChE) activity. Activity was measured by Ellman assay with acetylthiocholine iodide as substrate in 0.1 M phosphate buffer (pH 8.0).

**Figure 2:**
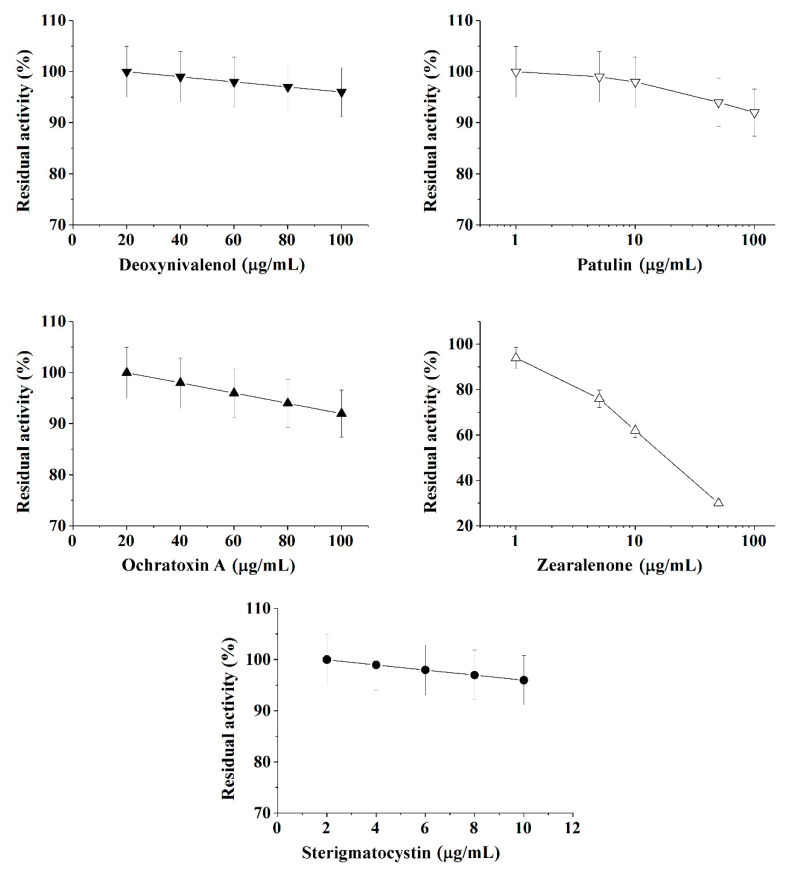
Inhibition effect of deoxynivalenol, patulin, ochratoxin A, zearalenone, and sterigmatocystinon on butyrylcholinesterase (BChE) activity. Activity was measured by Ellman assay with butyrylthiocholine iodide as substrate in 0.1 M phosphate buffer (pH 8.0).

**Figure 3:**
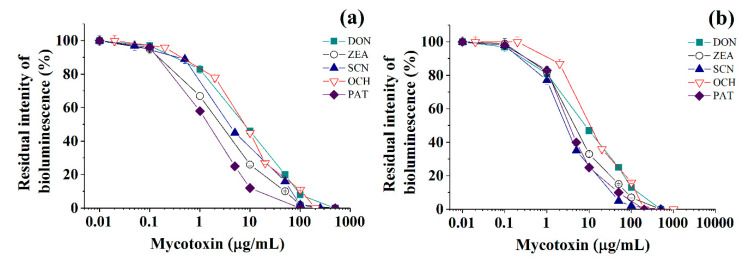
Residual intensity of luminescence of immobilized *Photobacterium* sp. 9.2 (**a**) and *Photobacterium* sp. 17 (**b**) cells in the presence of various mycotoxins (DON—deoxynivalenol, ZEA—zearalenone, SCN—sterigmatocystin, OCH—ochratoxin A, PAT—patulin) in discrete analysis.

It was shown with sufficiently good reproducibility, that cholinesterases and immobilized luminescent photobacterial cells can be successfully used to perform the quantitative express-analysis of at least one of five mycotoxins (deoxynivalenol, ochratoxin A, patulin, sterigmatocystin, and zearalenone) in liquid media in discrete mode (Table 1 and Figure 1, Figure 2 and Figure 3).

Calibration plots for quantitative analysis are presented in Figure 1, Figure 2 and Figure 3 in the coordinates, in which the obtained data can be successfully linearized. Enzymes were found to be less sensitive than photobacteria to the presence of mycotoxins. In general, a shift in the range of working concentrations upward was noted for enzymes. Both enzymes showed lower sensitivity to the presence of patulin as compared to cells.

AChE was more sensitive to ochratoxin A and sterigmatocystin and less sensitive to DON as compared to BChE. According to the results obtained, the adjusted coefficient of determination (R^2^) for both enzymes was close to 1 (Table 1), whereas for luminescent bacterial cells, mainly for the *Photobacterium* sp. 17, the values of R^2^ below 0.97 were obtained. Note that calculated R^2^ values exceeding 0.9 indicate the possibility of using the corresponding tool (both enzymes and photobacteria in our case) for analytical purposes.

The results obtained allow the selection of the most acceptable analytical method for an express analysis of each mycotoxin in terms of the working concentration range and the lower limit of detection (LOD) value.

Thus, *Photobacterium* sp. 17 cells provide the lowest LOD and a fairly wide working concentration range for the analysis of deoxynivalenol, whereas *Photobacterium* sp. 9.2 ensure the best set of parameters for ochratoxin A and zearalenone detection, *Photobacterium* sp. 17 cells are optimal for patulin, and AChE is the instrument of choice for sterigmatocystin.

### 2.2. Assessment of Toxicity of the Reaction Medium Obtained after Hydrolysis of Zearalenone by His_6_-OPH in the Media with Different pH

Using acetyl-cholinesterases, photobacterial cells, and ELISA Test Kit as analytical tools, we have proven the possibility of zearalenone destruction under the action of His_6_-OPH in a liquid medium at different pH values at an initial mycotoxin concentration of 65 ± 3 μg/mL (Table 2). The results of this study agree with those we obtained earlier [17]. It was noted that the low sensitivity of enzymes allowed the detection of only the initial concentration of mycotoxin in the test solution. The residual concentration of zearalenone after the action of His_6_-OPH in the case of enzymatic analytical agents could only be determined using BChE for a sample with pH 7.4 (Table 2). Photobacterial cells, however, ensured accuracy high enough to reliably assess the degree of zearalenone destruction under the action of His_6_-OPH for different medium pH values. The accuracy of mycotoxin detection with photobacteria was in fact found to be at least as high as that ensured with the ELISA Test Kit.

### 2.3. Zearalenone Biodegradation in Feed Grain Mixture under the Action of the Enzyme His_6_-OPH

In a model experiment using enzymes, photobacterial cells, and ELISA Test Kit as analytical agents, it was possible for the first time to assess detoxification of food raw materials (feed grain mixture) initially contaminated with zearalenone at a concentration of 10 mg/kg. Feed was treated with His_6_-OPH enzyme to push the toxin concentration below the levels specified in the generally accepted quality standards, so that the resulting contaminant concentration was below 1 mg/kg (Table 3).

In the case of enzymatic analytical agents, only BChE allowed the determination of the concentration of zearalenone in the analytical sample of the raw material with the maximum concentration of zearalenone (Table 3). The use of biological analytical agents resulted in slightly higher values of toxicant concentration than in case of using the ELISA Test Kit. This was probably due to the presence of substances other than zearalenone, which reduce the activity of enzymes in the analytical samples. These substances could have originated from the feedstock and been transferred together with zearalenone during the extraction stage. It is important that the calculated detoxification degree was similar in the case of photobacterial cells and ELISA Test Kit (Table 3).

Thus, photobacteria can be efficiently used as analytical agents for evaluating the detoxification of real raw materials. They can greatly facilitate the search for bio-destructors of any target mycotoxin, as well as the assessment of the efficiency of detoxification using various biological agents. It was shown that an acetonitrile-based extractant can be recommended for the extraction of zearalenone from the feedstock during sample preparation. This extractant provides a high degree of recovery of this toxicant (Table 3), which is consistent with the previously published data [22,23].

## 3. Discussion

The development of new express analytical methods that could reduce the time and cost of research is very topical. Novel efficient techniques are urgently needed both for selecting the potential bio-destructors of mycotoxins and for assessing the effectiveness of their action under various conditions. It was shown that cholinesterases and immobilized luminescent cells of photobacteria give a stable analytical signal in the presence of common mycotoxins (Table 1).

The key points for choosing a biological analytical agent for the determination of mycotoxins are LOD and working range. The availability of equipment, the duration of the analysis, the volume of the sample, the need to use additional reagents, and the range of analyzed mycotoxins should also be taken into account. The duration of the analysis (with calibration graph available) was ca. 2–5 min in the case of using enzymes in this work, and 30 min in that of photobacterial cells.

The volume of mycotoxin-containing sample sufficient for the contaminant determination was 10 μL and 4 μL in the cases of cholinesterases or luminescent bacteria, respectively. The analysis of mycotoxins was carried out using generally available laboratory equipment: a spectrophotometer (in the case of enzymes) or a luminometer (in the case of photobacteria). In contrast to the case of luminescent bacteria, the enzyme-based analysis required the use of additional reagents: acetylthiocholine iodide or butyryl-thiocholine iodide was used as substrate and 5,5′-dithiobis-(2-nitrobenzoic acid) was used as indicator.

The enzymes were found to be less sensitive to the presence of mycotoxins in the analyzed samples. This was probably due to the fact that in the luminescent cells, in addition to the main analytical luminescent system, there is a large number of interconnected enzymatic systems that are sensitive to the toxicant. Thus, in general, under the conditions of the experiments, the enzymatic analytical systems based on AChE and BChE are somewhat inferior in terms of significant parameters to the cells of photobacteria (Table 1).

Among the studied bacterial strains, despite the high degree of phylogenetic homology, the best analytical characteristics corresponded to *Photobacterium* sp. 9.2 cells (Table 1). The luminescent *Photobacterium* sp. 9.2 and *Photobacterium* sp. 17 cells ensured 44%–83% lower LOD values for zearalenone determination compared to the case of *Photobacterium phosphoreum* B-1717 [17], and for deoxynivalenol and sterigmatocystin the range of working concentrations was 1.5–3.5 times greater in this study. Calibration curves for the quantitative determination of patulin by using photobacterial cells immobilized in poly(vinyl alcohol) cryogel are presented in this work for the first time (Table 1).

The use of the studied biological analytical agents (enzymes and cells) specifically for the direct quantitative determination of mycotoxins in feed is most likely to be inappropriate. The reason is that the solvents that are used to extract mycotoxins into an analytical sample and ensure a high degree of contaminant recovery can themselves be toxic to living cells and enzymes. For an accurate quantitative analysis, in this case, complex sample preparation and standardization for the secondary toxicant are required; in this case, preference is given to liquid chromatography and ELISA [10].

It was shown in this work that the most expedient way to use the photobacteria is the express analysis of mycotoxins, which is an important stage in the assessment of the degree of detoxification of analytical samples due to the destruction of mycotoxins. The analytical samples often contain component toxic to cells other than mycotoxin, e.g., methanol or acetonitrile which are commonly used as extractants-solvents of mycotoxins. In this case it is necessary to account for these additional toxicants when carrying out the calculations, e.g., by appropriate normalization, which was also done in this work.

Despite their somewhat lower sensitivity, enzymes could be used in searching for effective bio-destructors of some mycotoxins. In this case the destruction products are the most predictable, and additional agents can be introduced (if necessary) into the reaction system for directed detoxification of the toxic intermediates of enzymatic mycotoxins’ decomposition [5].

The expediency of using the His_6_-OPH for the destruction of lactone-containing mycotoxins, like zearalenone, was confirmed in the work (Table 2 and Table 3). Using immobilized cells of photobacteria as analytical agents, it was shown for the first time that a shift in the pH of the medium from 7.4 to 8.5 when using His_6_-OPH makes it possible to improve the degree of zearalenone destruction from 93%–94% to 98%–99% (Table 2).

In general, the results obtained are consistent with the literature data on the relatively high hydrolytic activity of lactonases in relation to zearalenone in neutral and slightly alkaline media [17]; the catalytic activity of the enzyme decreases when the pH is lowered [12,18]. The established regularity, allows us to draw certain conclusions about the prospects of creating food supplements based on His_6_-OPH. The controlled release of this enzyme in the digestive system in the areas with alkaline pH values can ensure efficient decomposition of mycotoxins that enter the body of animals with nutrition under the action of His_6_-OPH. His_6_-OPH can be potentially introduced into the feed contaminated with zearalenone, as in the case of recombinant lactonohydrolase [12], for mycotoxin detoxification. Additionally, using His_6_-OPH in a medium at pH 8.5 provided up to five times faster degradation of zearalenone as compared to recombinant lactonohydrolase expressed in *Penicillium canescence*. This was also demonstrated in this work for the first time.

There is a general consensus that bioanalytical agents such as cholinesterases and photobacterial cells are not selective in their inhibition reactions with various toxins. However, in this work, some obvious preferences were found for bacterial strains, possessing close phylogenetic relations, and cholinesterases in reactions with the same mycotoxins. The best bioindicators in terms of sensitivity and working range (μg /mL) were determined as follows: *Photobacterium* sp. 17 cells for analysis of deoxynivalenol (0.8–89) and patulin (0.2–32); *Photobacterium* sp. 9.2 cells-for analysis of ochratoxin A (0.4–72) and zearalenone (0.2–32); AChE for analysis of sterigmatocystin (0.12–219). Cholinesterases were found to be less sensitive than cells. Calibrations for quantitative determination of patulin using immobilized photobacteria are presented in this work for the first time.

Generally, the use of luminescent cells can significantly reduce the time and financial costs when conducting primary evaluative analyzes of the mycotoxin content in samples during laboratory studies. The efficiency of enzymatic destructors in reactions with mycotoxins can be adequately evaluated using simple equipment and a well-known approach. The information obtained regarding the preferences of bioanalytical agents used for the analysis of a particular mycotoxin will be useful for those researchers who are engaged in the scientific search for bio-destructors of mycotoxins, and simplifies their laboratory screening.

## 4. Materials and Methods

### 4.1. Chemicals and Strains

Mycotoxins (ochratoxin A, sterigmatocystin, zearalenone, deoxynivalenol, and patulin); cholinesterase enzymes (AChE and BChE); 5,5′-dithiobis (2-nitrobenzoic acid), acetylthiocholine iodide, and butyrylthiocholine iodide were purchased from Sigma-Aldrich (St. Louis, MO, USA). For the experiments, concentrated solutions of mycotoxins in methanol were preliminarily prepared. Solutions of mycotoxins of the required concentration were prepared by diluting the original stock solutions of mycotoxins in methanol. In the analysis, the quenching of the bioluminescence of the immobilized luminous bacteria under the action of the methanol present in the reaction medium was taken into account. Poly(vinyl alcohol) 16/1 (M.w. 84 kDa) was purchased from Sinopec Corp (Beijing, China); peptone and yeast extract were purchased from Difco (Becton, Dickinson and Company, Franklin Lakes, NJ, USA); inorganic salts for Farghaly growth medium and other reagents were purchased from Chimmed (Moscow, Russia). *Photobacterium* sp. 9.2 and *Photobacterium* sp. 17 were provided by A.D. Ismailov (Lomonosov Moscow State University, Moscow, Russia).

### 4.2. Growth Cells Conditions, Immobilization and Luminescence Measurements

*Phosphoreum* sp. cells were grown in the Farghaly growth medium and maintained in a submerged culture at 18 °C at 60 rpm (IRC-1-U temperature-controlled shaker, Adolf Kuhner AG Apparatebau, Switzerland). The optical density of the culture medium was determined by spectrophotometry at 660 nm (Agilent UV-853 spectrophotometer, Agilent Technologies, Waldbronn, Germany), and the cells were cultivated for 22 h to an optical density of 0.73 ± 0.05, separated from the culture medium by centrifugation (5000 rpm, 15 min, J2 21 centrifuge, Beckman, Brea, CA, USA), and cell biomass used in the immobilization procedure. The procedure for immobilizing the bioluminescent cells in poly (vinyl alcohol) (PVA) cryogel was described previously [20]. The cell biomass was mixed with a 10% (*w*/*v*) aqueous PVA solution to obtain a 10% (*w*/*w*) concentration of bacterial cells. This mixture was pipetted into 96-well microplates (0.2 mL/well), which were placed in a freezer at −20 °C for 24 h and then thawed at +4 °C. The cylinder granules of PVA cryogel (d = 6.6 ± 0.1 mm, h = 4.8 ± 0.1 mm) formed in this way contained cells immobilized by inclusion. The average wet weight of one granule was 0.172 ± 0.001 g.

Luminescence of immobilized bacteria was measured using a 3560 microluminometer (New Horizons Diagnostics Co, Columbia, MD, USA). Luminescence detection was performed in aqueous media based on a 2% NaCl solution at 10 ± 1 °C. The maximum level of luminescence (I_0_) was determined for 10 s at 10 °C after thermal equilibration of the flow-through system. For practical purposes, the residual intensity of luminescence was used (I/I_0_), which was expressed as a percentage of the baseline signal (I_0_). The residual intensity of luminescence (I/I_0_) was analyzed in a discrete test after the exposure of the cells to a certain mycotoxin for 0.5 h after its addition to medium containing the analytical agent. The assays were performed in triplicate.

### 4.3. Mycotoxins Analyses with Cholinesterase Enzymes (AChE and BChE)

The activity of cholinesterases was determined using the Ellman method [24]. Briefly, 0.96 mL of 0.1 M phosphate buffer (pH 8.0) in a spectrophotometric cell was supplemented with 10 μL of 20 mM 5,5′-dithiobis (2-nitrobenzoic acid) in a 0.1 M phosphate buffer (pH 7.0), containing 1.5 g/L Na_2_CO_3_. Then, 10 μL of 0.01 mg/mL AChE or 0.2 mg/mL BChE followed by a 10 μL of 0–10 mg/mL mycotoxin in methanol or ethanol was added and vigorously mixed. Reaction was initiated by addition of 10 μL of 50 mM acetylthiocholine iodide or 200 mM butyrylthiocholine iodide for AChE or BChE, respectively.

The rate of formation of 2-nitro-5-thiobenzoic acid at λ = 412 nm was determined using the Agilent 8453 UV-visible spectroscopy system (Agilent Technologies, Waldbronn, Germany). All enzymes, substrates, mycotoxins, and other reagents were freshly prepared before use.

Enzyme activity without any toxins or solvents was monitored, and results were adjusted accordingly. One unit of AChE or BChE activity was defined as the enzyme amount that hydrolyzed 1 μmol of substrate per min at 25 °C. The experiments were realized in triplicate.

### 4.4. Hydrolysis of Zearalenone in Medium with Different pH under the Action of the His_6_-OPH

For the experiment, the initial concentration of zearalenone in the reaction medium based on phosphate buffer (pH 7.4 or 8.5) was 65 ± 3 mg/L. The initial toxicity of this solution was evaluated under the conditions indicated above. The solution of the His_6_-OPH (0.1 mg/mL) with an activity of 200 U/mL was added to zearalenone solution.

The treatment of the mycotoxin was carried out for 1 h at room temperature without agitation, and the residual toxicity of the obtained solution was verified using immobilized luminescent cells or cholinesterases in a discrete mode of analysis (Table 2).

### 4.5. Hydrolysis of Zearalenone in Feed Grain Mixture under the Action of the His_6_-OPH

Using 0.1 M phosphate buffer (pH 7.5), a solution of zearalenone with concentration 2 g/L was prepared from its concentrated methanol stock solution. The preparation was injected (in the form of a spray) into the feed grain mixture for rats using Classic TiTBiT (Dmitrov, Moscow Region, Russia) at the rate of 10 mg of zearalenone per 1 kg of grain mixture. After that, one half of the grain mixture containing zearalenone was sprayed with a solution of His_6_-OPH, prepared based on 0.1 M phosphate buffer (pH 7.5), at a dose of 4000 U/kg of the grain mixture, after which the mixture was mechanically stirred and kept for 12 h at 25 °C.

The procedure for enzyme production and purification was detailed previously [25]. The activity of His_6_-OPH was determined as described previously [26], with 7.8 mM aqueous Paraoxon stock solution at 405 nm using the Agilent 8453 UV-visible spectroscopy system (Agilent Technology, Waldbronn, Germany) equipped with a thermostated analytical cell.

After 12 h, the zearalenone concentration was determined in the feed samples with zearalenone, pretreated with the enzyme or without the pretreatment, as well as in the control sample, without any additives. 15 g of each feed grain mixture sample was ground into powder in a laboratory mill and was subject to triple extraction with 40 mL of 84% acetonitrile aqueous solution (*v*/*v*) by mechanical shaker for 15 min. Fractions obtained from each sample were pooled. Then the extract was filtered through paper filters and evaporated to dryness under nitrogen flow. The obtained weighed portion was dissolved in 300 μL of methanol. A total of three samples of methanol stock solution from grain mixture samples were obtained (Table 3). Next, the required dilution of samples was carried out using aqueous buffer solutions to a methanol content of no more than 2%–4%, and the concentration of zearalenone in the samples was determined using cholinesterase enzymes, luminescent cells, or ELISA kit (Table 3).

### 4.6. Determination of Zearalenone by Enzyme-Linked Immunosorbent Assay (ELISA) Test Kit

Analyses were carried out using MaxSignal^®^ Zearalenone ELISA Test Kit (Bio Scientific Corp, Austin, TX, USA) with sensitivity 0.3 ng/mL Samples were prepared according to the instructions provided by the manufacturers of the ELISA kits. Optical density was measured at 450 nm using a microplate reader iMark (Bio-Rad Laboratories, Inc., Hercules, CA, USA).

### 4.7. Calculations

The data were linearized in various plots with OriginPro (ver. 9.4.2, OriginLab Corporation, Northhampton, MA, USA) and the most suitable coordinates were selected. The limit of detection (LOD) and limit of quantification (LOQ) for enzymes and photobacterial cells were calculated as a minimal mycotoxin concentration which is distinguishable from the blank measurement (i.e., enzymes or cells without inhibitors) by more than three sigmas and ten sigmas (standard deviation, σ) in at least, six independent measurements), respectively. All measurements with MaxSignal^®^ Zearalenone ELISA Test Kit were repeated three times, and the results were analyzed with the Microplate Manager^®^ 6, version 6.3.

The data are presented as means ± standard deviation (±σ) unless otherwise stated.

## 5. Patents

RU Patent #2394910 Luminescent biocatalyst for the determination of toxicants.

RU Patent #2255975 Recombinant plasmid DNA pTES-His-OPH and producer of oligo-histidine containing organophosphate hydrolase.

## Figures and Tables

**Table 1:** Analytical characteristics of different mycotoxins’ assay (Figure 1, Figure 2 and Figure 3) based on application of cholinesterases (Figure 1 and Figure 2) or immobilized luminescent cells (Figure 3) and linearization equations (R^2^ is adjusted coefficient of determination).

Mycotoxin	Coefficients of the Linearization Equation		R^2^	Working Range, µg/mL	Limit of Quantification (LOQ), µg/mL	Limit of Detection (LOD), µg/mL
	a	b				
AChE						
Deoxynivalenol ^1^	97.9 ± 0.1	4.0 ± 0.1	0.998	≥1698	1698	563
Ochratoxin A ^1^	64.9 ± 5.4	180.9 ± 9.9	0.979	30−354	30	10
Patulin ^1^	98.9 ± 0.1	4.0 ± 0.1	0.998	≥2951	2951	984
Sterigmatocystin ^1^	65.3 ± 0.2	21.5 ± 6.0	0.999	0.12−219	0.12	0.04
Zearalenone ^2^	111.5 ± 0.9	0.9 ± 0.01	0.999	29−103	29	10
BChE						
Deoxynivalenol ^2^	101.0 ± 0.1	0.05 ± 0.001	0.999	320−1720	320	105
Ochratoxin A ^2^	102.0 ± 0.1	0.1 ± 0.01	0.999	170−870	170	56
Patulin ^1^	104.0 ± 0.4	6.0 ± 0.2	0.997	≥1548	1548	511
Sterigmatocystin ^1^	101.0 ± 0.1	0.5 ± 0.01	0.999	32−172	32	11
Zearalenone ^1^	108.0 ± 0.2	45.9 ± 0.1	0.999	3−107	3	1
*Photobacterium* sp. 9.2 cells						
Deoxynivalenol ^1^	84.1 ± 1.2	38.0 ± 0.6	0.999	1−66	1	0.3
Ochratoxin A ^1^	73.6 ± 10.2	31.5 ± 5.5	0.923	0.4−72	0.4	0.13
Patulin ^1^	57.1 ± 1.3	45.2 ± 1.4	0.999	0.3−8	0.3	0.1
Sterigmatocystin ^1^	71.8 ± 6.3	33.0 ± 3.9	0.981	0.4−52	0.4	0.13
Zearalenone ^1^	57.6 ± 9.0	28.2 ± 5.6	0.934	0.2−32	0.2	0.07
*Photobacterium* sp. 17 cells						
Deoxynivalenol ^1^	82.1 ± 2.6	34.4 ± 1.4	0.995	0.8−89	0.8	0.27
Ochratoxin A ^1^	87.0 ± 13.3	35.8 ± 7.1	0.925	1.1−102	1.1	0.37
Patulin ^1^	60.2 ± 10.1	29.9 ± 6.4	0.912	0.2−32	0.4	0.07
Sterigmatocystin ^1^	63.8 ± 10.2	34.7 ± 6.1	0.941	0.3−25	0.4	0.1
Zearalenone ^1^	70.5 ± 11.6	33.0 ± 7.4	0.905	0.4−48	0.4	0.13

^1^ Activity = a − bx lg (concentration, µg/mL); ^2^ Activity = a − bx (concentration, µg/mL).

**Table 2:** The residual zearalenone concentrations in the media with hexa-histidine-tagged organophosphorus hydrolase (His_6_-OPH) after 1 h of enzymatic treatment. The initial zearalenone concentration was 65 ± 3 μg/mL.

Analytical Method *	Zearalenone, μg/mL		* DH, %	Reference
	pH 7.4	pH 8.5	pH 8.5	
BChE	3.8 ± 0.1	-	-	This work
*Photobacterium* sp. 9.2	4.3 ± 0.1	0.67 ± 0.03	98.8 ± 0.9	This work
*Photobacterium* sp. 17	4.5 ± 0.1	0.63 ± 0.03	99.0 ± 0.9	This work
MaxSignal^®^ Zearalenone ELISA Test Kit	3.8 ± 0.1	0.58 ± 0.02	99.1 ± 0.9	This work
	3.9 ± 0.2	-	-	[17]
*Photobacterium phosphoreum* B-1717	4.1 ± 0.2	-	-	[17]

* DH is the degree of zearalenone hydrolysis = the percentage of the hydrolyzed zearalenone concentration in relation to its initial level; -: samples were not analyzed.

**Table 3:** Residual concentrations of zearalenone in the feed grain mixture (initial contamination of feed grain mixture by zearalenone was 10 mg/kg) after its treatment with enzyme His_6_-OPH (ED) during 12 h and without it (NE).

Analytical Method	Zearalenone, mg/kg Feed		* R, %	** D, %
	NE	ED		
BChE	8.5 ± 0.3	***	85 ± 4	***
*Photobacterium* sp. 9.2	8.1 ± 0.3	0.82 ± 0.04	81 ± 4	89.9 ± 4.1
*Photobacterium* sp. 17	8.2 ± 0.3	0.85 ± 0.05	82 ± 4	89.6 ± 4.2
MaxSignal^®^ Zearalenone ELISA Test Kit	7.9 ± 0.3	0.77 ± 0.03	79 ± 4	90.3 ± 4.1

* R is the degree of zearalenone recovery; the percentage of the NE zearalenone concentration to its initial concentration; ** D is the level of zearalenone detoxification in feed; the percentage of the change in zearalenone concentration in feed grain mixture as the result of enzymatic destruction (NE-ED) to the non-enzymatic degradation (NE); *** There was no inhibition of BChE activity.

## Data Availability

The data presented in this study are available in this article.
